# Intraoperative Peritoneal Interleukin-6 Concentration Changes in Relation to the High-Mobility Group Protein B1 and Heat Shock Protein 70 Levels in Children Undergoing Cholecystectomy

**DOI:** 10.1155/2020/9613105

**Published:** 2020-07-08

**Authors:** Marzena Tylicka, Ewa Matuszczak, Joanna Kamińska, Wojciech Dębek, Beata Modzelewska, Tomasz Kleszczewski, Violetta Dymicka-Piekarska, Joanna Matowicka-Karna, Maria Karpińska, Olga M. Koper-Lenkiewicz

**Affiliations:** ^1^Department of Biophysics, Medical University of Białystok, Mickiewicza 2a, 15-089 Białystok, Poland; ^2^Department of Pediatric Surgery and Urology, Medical University of Białystok, Waszyngtona 17, 15-274 Białystok, Poland; ^3^Department of Clinical Laboratory Diagnostics, Medical University of Białystok, Waszyngtona 15A, 15-269 Białystok, Poland

## Abstract

The aim was the evaluation of IL-6 concentration in peritoneal lavage fluid of children which underwent cholecystectomy to ascertain if there is a difference in early inflammatory response depending on the type of surgical approach (open vs. laparoscopy). The analysis of high-mobility group protein B1 (HMGB1) and heat shock protein 70 (HSP70) was performed to find out if the source of IL-6 was related to tissue damage. IL-6 concentration in peritoneal lavage fluid samples, obtained at the beginning and at the end of the laparoscopic (*N* = 23) and open cholecystectomy (*N* = 14), was tested with a routinely used electrochemiluminescence assay. The concentrations of HMGB1 and HSP70 were analyzed with the use of an ELISA method. Statistical analysis was performed using the STATISTICA PL release 12.5 Program. The differences were assessed using the Mann-Whitney *U* test and Wilcoxon matched pairs test. Correlations were studied by using the Spearman correlation test. Our results demonstrated significant peritoneal lavage fluid IL-6 concentration growth measured at the end of the cholecystectomy as compared to the beginning, regardless of the type of the procedure. IL-6 growth during open cholecystectomy was greater compared to laparoscopic cholecystectomy (62.51-fold vs. 3.19-fold). IL-6 concentration did not correlate with HMGB1 and HSP70, which indicate that the significant growth of this cytokine was not related to mechanical tissue damage due to surgical procedure. A clinical significance of the study could be related to the fact that the evaluation of IL-6 concentration in peritoneal lavage fluid may be useful to assess an early local inflammatory response.

## 1. Introduction

Cholecystectomy is a common surgery performed using the open or laparoscopic approach. It is established that the laparoscopic cholecystectomy is more beneficial compared to the open surgery procedure [1–4]. On the other hand, in the available literature, there are some voices indicating laparoscopic intervention as equivalent or even inferior compared to open surgery [5]. Despite the type of the surgery, both operations include mechanical tissue injury, local hemorrhage, ischemia, ischemia-cellular stress, and hypoxia-reperfusion injury which altogether cause the activation of innate and adaptive immunity. The most common implications of these events are impaired wound healing and infections leading to longer hospitalization time, impaired long-term cognitive functions, and increased mortality [6].

IL-6 is the main cytokine produced in response to inflammatory agents and tissue injury [7, 8]. It is produced by different types of cells: monocytes/macrophages, T cells, epithelial cells, stromal cells, muscle cells, and hematopoietic cells [9, 10]. During abdominal operation, peritoneal macrophages are activated and release proinflammatory cytokines (including IL-6) into the peritoneal fluid, which may promote adhesion formation [11]. Studies of Saba et al. [12] on a mouse model reveal that selective immunosuppression, using IL-6 neutralizing antibodies preoperatively, leads to a reduction of adhesion formation. Cheong et al. [11] highlighted the importance of inflammatory cytokine concentration evaluation in peritoneal lavage fluid immediately after the operation.

Damage-associated molecular patterns (DAMPs) are released from dying or damaged cells occurring as a result of so-called “sterile inflammation,” caused, i.e., by trauma, burn, or ischemia [7, 13]. Among DAMPs, which are able to directly and indirectly promote inflammatory process, we can distinguish high-mobility group protein B1 (HMGB1), heat shock proteins (HSPs), mitochondrial (mt) DNA, and others [14, 15]. Study of Bianchi [16] shows that mtDNA leads to Toll-like receptor (TLR) 9 and NF-kappa beta (NF-*κ*B) activation, which is recognized as a transcription factor regulating human *IL-6* gene transcription, as *cis*-regulatory elements in the *IL-6* gene 5′ flanking region have binding sites for NF-*κ*B [7].

Currently, in routine laboratory diagnostics, inflammatory process after surgery can be monitored by the white blood cell count (WBC) and C-reactive protein (CRP) concentration evaluation [6, 17]. Literature data indicates that the levels of circulating plasma proinflammatory cytokines, including IL-6, may be related to the extent and severity of surgical procedure [18–22]. Moreover, available literature indicates that peak in IL-6 production or messenger RNA (mRNA) *IL-6* and their kinetics differ depending on causative agent and research model (e.g., human/animal model, type of sample: serum, plasma, urine, whole blood, adipose tissue, skeletal muscle) [23–32]. The intraoperative peritoneal fluid IL-6 concentration changes in open versus laparoscopic cholecystectomy have not been widely studied so far. Thus, the aim of the study was the evaluation of IL-6 concentration in peritoneal lavage fluid of children, which underwent cholecystectomy to find out a difference in early local inflammatory response depending on the type of intervention (laparoscopy or open). Furthermore, we analyzed concentrations of selected DAMPs (HMGB1 and HSP70) to assess if the source of IL-6 was related to mechanical tissue damage.

## 2. Material and Methods

### 2.1. Patients

Thirty-seven of forty children, who were admitted between 2013 and 2017 to the Pediatric Surgery Department of the Medical University of Bialystok for possible cholecystectomy, were included in the study. Inclusion criterion was patients' age between 4 and 17 years. Study group was divided by taking into consideration the procedure used to perform cholecystectomy: open surgery group (*N* = 14) and laparoscopic surgery group (*N* = 23).

Criteria for performing laparoscopic cholecystectomy were as follows: (a) age > 5 years, (b) low risk for common bile duct (CBD) stones, as defined by normal liver enzymes and common bile duct diameter of 5 mm or less, (c) elective operation, (d) absence of major medical problems, and (e) no previous upper abdominal operations. Criteria for performing open cholecystectomy were age < 5 years and previous abdominal operations which prevent safe abdominal access.

Three children were excluded from the analysis because of exclusion criteria: oncological history, severe existing infections, and immunological or cardiovascular diseases that required long-term medication. Those patients, whose parents gave informed consent for both clinical and biochemical follow-ups, were included in the study.

During cholecystectomy, intraoperative drainage of the peritoneal cavity with routinely used normal saline (0.9% NaCl) was performed. Normal saline has gained wide popularity in abdominal surgery and is used to remove cells, tissue debris, or blood clots. All patients at the beginning and at the end of the laparoscopic and open cholecystectomy underwent peritoneal lavage. Immediately after incision (at the beginning) and before closure (at the end), normal saline was poured into the peritoneal cavity and was aspirated back into a syringe. The amount of 0.9% NaCl solution applied to the lavage of the peritoneum was 3.5 mL per kilogram of the patient's body mass (Me = 189 mL; IQs: 49.00-210.00 mL), of which about 90% of fluid was aspirated back (during surgery condition, it is not possible to aspirate back the whole volume of the liquid). 10 mL each of liquid aspirated from the peritoneal lavage at the beginning and at the end of surgery was taken to the experiment.

Patients received standard surgical care and postoperative treatment after surgical approach according to the standard treatment protocols of our clinic and were given intravenous paracetamol after the surgeries which does not suppress the inflammation [33].

Approval for this study was obtained from the Local Bioethics Committee (Permission No.: R-I-002/471/2018). Procedures were in accordance with the ethical standards set by the Declaration of Helsinki given by the World Medical Association.

### 2.2. IL-6, HMGB1, and HSP70 Concentrations' Measurement

The concentration of IL-6 in peritoneal lavage fluid was tested on the COBAS e411 immunochemical analyzer (Roche Diagnostics) by means of IVD (*in vitro diagnostics*) test using the electrochemiluminescence immunoassay (ECLIA) method. Peritoneal lavage fluid samples were not diluted prior to analysis. The assay range was to equal 1.5-5000 pg/mL (determined by the lower limit of detection and the highest point of the standard curve). According to the manufacturer's instructions, the following cytokines IL-1*α*, IL-1*β*, IL-2, IL-3, IL-4, IL-8, interferon-*γ*, and tumor necrosis factor-*α* in the concentration of 50 ng/mL do not show cross reactions.

The concentrations of high-mobility group protein B1 (HMGB1) and heat shock protein 70 (HSP70) in peritoneal lavage fluid samples were analyzed with the use of an ELISA (enzyme-linked immunosorbent assay) method, as described elsewhere [34].

HMGB1 concentration was measured using an EIAB Human HMGB1/high-mobility group protein B1 ELISA kit (Catalogue No. E0399h) in compliance with the manufacturer's instructions. Peritoneal lavage fluid samples were not diluted prior to analysis. The detection range for the kit is between 0.156 and 10.00 ng/mL. According to the manufacturer's instruction, the sensitivity (minimum detectable dose) of the assay kit is 0.1 ng/mL and it is determined experimentally by adding three standard deviations to the average optical density (OD) of twenty replicates of the zero standard and calculating the corresponding concentration. This illustrates the lowest concentration distinguishable from zero. Values < 0.1 ng/mL were considered as 0.00 ng/mL. In the current study, we did not obtain values above the upper limit range.

HSP70 concentration was evaluated using a Biorbyt Human heat shock proteins (HSP70) ELISA kit (Catalogue No. Orb397059) in compliance with the manufacturer's instructions. The detection range for the kit is between 125 and 8000 pg/mL. In the first experiment, peritoneal lavage fluid samples were not diluted prior to analysis. In this experiment, we received 36 results above the ELISA kit detection range (8000 pg/mL). Therefore, we repeated the experiment with 50-fold diluted peritoneal lavage fluid samples. Obtained results were multiplied by the dilution factor.

According to the manufacturer's instruction, the sensitivity (minimum detectable dose) of the assay kit for HSP70 is 60 pg/mL and it is determined experimentally by adding three standard deviations to the average optical density (OD) of twenty replicates of the zero standard and calculating the corresponding concentration. This illustrates the lowest concentration distinguishable from zero. Values < 60 pg/mL were considered as 0.00 ng/mL.

### 2.3. CRP and WBC Evaluation

During infection or inflammation C-reactive protein (CRP) concentration rises rapidly within the first 6 to 8 hours, while the peak is observed after 48 hours [35]. Therefore, patients' blood for CRP evaluation was collected twice: about 2 hours prior to surgery and 8-10 hours after conducted procedure. The same was done for white blood cell count (WBC) evaluation. In order to determine the CRP, the blood was collected into the test tubes with clotting activator (S-Monovette, SARSTEDT, 1.2 mL). Within 2 hours after the venipuncture, the blood was centrifuged to obtain serum samples and CRP was tested on the Cobas C501 (Roche Diagnostics) analyzer. In order to determine WBC on a XT 4000i (Sysmex) hematological analyzer, the blood was collected into the test tubes with EDTA-K_3_ (S-Monovette, SARSTEDT, 1.2 mL).

### 2.4. Data Analysis

Statistical analysis was performed using the STATISTICA PL release 12.5 Program. All the results are presented as median (Me) within the 25^th^ and 75^th^ percentiles (IQs). Data normality was analyzed using the Shapiro-Wilk test, which proved that IL-6 concentration in the peritoneal lavage fluid did not follow the normal distribution. Therefore, the differences in continuous variable values were assessed using the Mann-Whitney *U* test and Wilcoxon matched pairs test which was used to test for significant differences between two conditions of an independent variable in an experiment where the same participants are responding in both groups (at the beginning and at the end of the surgery). Differences in baseline characteristics between two study groups were tested using the chi-square and Fisher exact test (categorical variables) or two-sample *t*-test (continuous variables) (Table 1). Correlations were studied by using Spearman's correlation test. Spearman's correlation coefficient *r* was used to assess the correlation strength. All tests were two-sided with a *p* = 5% level of statistical significance (*p* < 0.05).

## 3. Results

Peritoneal lavage fluid samples were obtained from all children at the beginning and at the end of the laparoscopic and open cholecystectomy procedures.

For each patient, key data such as type of surgical procedure, surgery duration, surgical findings, and the presence of any different pathology and demographic data included age and gender are collected and summarized in Table 1 with *p* values describing the comparability between two study groups. There were no significant differences between variables (*p* > 0.05) except for surgery duration and length of hospital stay after surgery for children undergoing laparoscopic and open cholecystectomy (*p* < 0.05).

### 3.1. Peritoneal Lavage Fluid IL-6 Concentration Results

The IL-6 growth during open cholecystectomy was greater compared to laparoscopic cholecystectomy (62.51-fold vs. 3.19-fold). In the case of both laparoscopic and open cholecystectomy procedures, peritoneal lavage fluid IL-6 concentration was significantly higher measured at the end of the operation than at the beginning (*p* < 0.05, Table 2).

**Figure 1:**
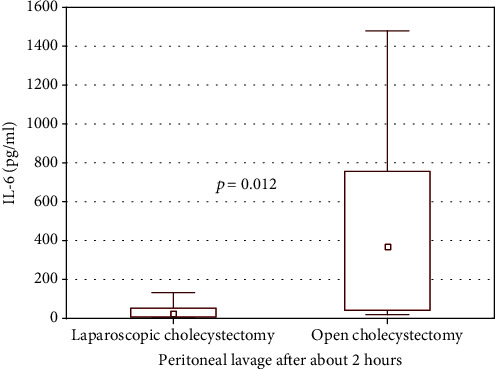
The comparison of IL-6 concentration in peritoneal lavage fluid of children underwent cholecystectomy after about 2 hours from the beginning of the laparoscopic (*N* = 12) compared to open procedure (*N* = 10).

Peritoneal lavage fluid IL-6 concentration strongly, positively correlated with the duration time of open cholecystectomy (*r* = 0.610; *p* = 0.021) as well as very strongly, positively correlated with the duration time of laparoscopic cholecystectomy (*r* = 0.744; *p* = 0.000). Thus, in the next step, we compared IL-6 concentration at the end of both types of cholecystectomy with comparable duration time of the procedure. Much higher IL-6 growth was observed at the end of open cholecystectomy lasting for about 2 hours (*N* = 10) compared to the end of laparoscopic surgery with comparable duration time (*N* = 12) (Figure 1). In the whole group of cholecystectomy children, IL-6 concentration strongly, positively correlated with the length of the hospitalization (*r* = 0.775; *p* = 0.000).

### 3.2. Peritoneal Lavage Fluid HMGB1 and HSP70 Concentration Results

In laparoscopic surgery, peritoneal lavage fluid HMGB1 concentration was significantly higher when measured at the end of the operation than at the beginning (*p* < 0.05). In the group of open cholecystectomy children, HMGB1 concentration was almost the same at the beginning compared to the end of the procedure (*p* > 0.05) (Table 3). In both surgery types, peritoneal lavage fluid HSP70 concentration was significantly higher measured at the end of the operation than at the beginning (*p* < 0.05, Table 3). Both HMGB1 and HSP70 concentrations, at the beginning and at the end of the procedures, did not correlate with IL-6 concentration (*p* > 0.05, respectively).

### 3.3. Blood CRP Concentration and WBC Results

Both CRP concentration and WBC in the blood were significantly higher 8-10 hours after applied procedures (laparoscopy and open surgery) as compared to the values obtained 2 hours prior to surgery (Table 4). Our study showed that CRP concentration measured after the open cholecystectomy increased 112.3-fold compared to the presurgery state. In the case of laparoscopic approach, 7.1-fold growth was observed. WBC value measured after the open cholecystectomy approach increased 1.7-fold compared to the initial state. In the case of laparoscopic surgery, growth was 1.5-fold.

## 4. Discussion

Observations based on this study are the first to show that a rapid and significant peritoneal lavage fluid IL-6 growth is already taking place during both laparoscopic and open cholecystectomy. However, IL-6 growth during open cholecystectomy was greater compared to laparoscopic cholecystectomy (62.51-fold vs. 3.19-fold). Our results are in line with other studies indicating increased inflammatory response in open surgery in comparison to the equivalent laparoscopic one [19–22, 36, 37].

This is also the first study which measured IL-6 concentration in peritoneal lavage fluid, obtained during cholecystectomy. Moreover, it should be noted that IL-6 concentration was measured on immunochemical analyzer used within routine laboratory diagnostics.

Numerous studies show that levels of circulating plasma proinflammatory cytokines, including IL-6, are related to the extent and severity of surgical procedure [18–22]. IL-6 concentration was mostly measured in plasma or serum [18–20]; rarely, it was assessed in peritoneum [19]. Even if the authors present results of the IL-6 concentration in peritoneal lavage fluid, data concern the postsurgery IL-6 evaluation [20, 36–38]. On the contrary, our measurements were conducted twice: at the beginning and at the end of cholecystectomy. Kumagai et al. [19] and Sammour et al. [36] postulate that IL-6 is a reasonable and reliable parameter for assessment of surgical stress up to 24 hours after surgery, because during this time it is mostly released into the peritoneal cavity. Zhang et al. [39] found that IL-6 is increased within 6 hours after surgery.

In the current study, we demonstrated that IL-6 concentration in peritoneal lavage fluid grows in the first hours of the operation; however, the growth in open procedure was much higher compared to laparoscopy. This subject has not been widely studied so far, as only Kawashima et al. [40] showed that the concentration of IL-6 in peritoneal lavage fluid was 11-fold higher at the end of the operation than immediately after incision at the beginning of the surgery. In our opinion, the difference regarding IL-6 results (11-fold growth vs. 3.19-fold growth) between Kawashima et al. and our study may result from (1) different methods applied for IL-6 evaluation (Kawashima et al. used the multiplex Bio-Plex Suspension Array System) (Bio-Rad Japan, Tokyo, Japan); multiplex method evaluating many ligands simultaneously in the same sample can lead to a cross reaction [41]; we chose the IVD test using electrochemiluminescence immunoassay (ECLIA) method (Roche Diagnostics); (2) different procedures being evaluated (Kawashima et al. studied IL-6 concentration in peritoneal cavity only during laparotomy; we analyzed both procedures, laparoscopy and open surgery); (3) different numbers of cases (Kawashima et al. *N* = 7, our group *N* = 23 and *N* = 14); (4) different studied groups (adults versus children and cancer patients versus nontumoral individuals).

A few earlier findings suggest that IL-6 concentration correlates with clinical parameters including length of surgery and length of hospitalization [19, 38, 42]. This was also confirmed by our study, as peritoneal lavage fluid IL-6 concentration is strongly, positively correlated with the duration time of cholecystectomy. Moreover, we noted a significant difference in IL-6 concentration between patients that underwent laparoscopic and open cholecystectomy just about 2 hours from the beginning of the operation. This is in line with findings of Shietroma et al. [43, 44], who observed that blood IL-6 concentration began to significantly increase as early as 1 hour from the beginning of the operation. Current results also confirmed the concept, presented by other authors [19, 36, 38, 42], that IL-6 concentration is very strongly and positively correlated with the hospital stay after surgery.

Our previous experiment showed that open surgical procedure is connected with more invasive tissue damage and in consequence with stronger inflammatory response compared to laparoscopic one [45]. Thus, in the next step, we analyzed the peritoneal lavage fluid concentrations of selected DAMPs: HMGB1 and HSP70 to find out if the source of IL-6 was related to the tissue damage. In our study IL-6 concentration did not correlate neither with HMGB1 nor with HSP70 concentration, which indicates that the significant growth of this protein was not related to surgical tissue injury.

Recently, HMGB1 has been identified as a mediator of lethal systemic inflammation and local inflammation [46]. Studies show that HMGB1 release by activated macrophages is created by active translocation from the nucleus to the cytoplasm, rather than by cell death [47–49]. HMGB1 can also be released by damaged or necrotic cells as a result of tissue injury or trauma [46]. In our study, a significant difference in HMGB1 concentration between beginning versus the end of the procedure was found only in laparoscopic cholecystectomy. Concentration of this protein in the case of open surgery was higher already after incision but did not change during operation. An explanation of our current findings could be delayed HMGB1 kinetics during the immune response [50], as mice exposed to endotoxin did not have detectable blood HMGB1 concentrations before 8 hours [47]. Moreover, Maca et al. [51] found that, except for HMGB1, the levels of other circulating DAMPs were higher in the group of major abdominal surgery patients as compared to the nonsurgical group. Thus, we conclude that peritoneal lavage fluid HMGB1 concentration measurement in children undergoing cholecystectomy would not be useful to evaluate an early immune response to tissue damage during surgery.

Heat shock proteins (HSPs), commonly referred to as “stress proteins,” provide protection against cellular and environmental stress factors [52, 53]. Extracellular or membrane-bound HSP70 and HSP90 have the ability to affect the release of cytokines, including IL-6 [54–56]. In our study, peritoneal lavage fluid HSP70 concentration was significantly higher when measured at the end of the operation than at the beginning, in case of both surgery types (laparoscopy and open surgery). Indeed, Mendoza-Sagaon et al. [57] indicate that there are no significant differences between laparoscopy and open cholecystectomy in terms of induction of the stress response, as steady-state mRNA levels of HSP70 were not significantly changed after both surgeries. However, it should be mentioned that their study was conducted on liver biopsies obtained from pigs. According to our best knowledge, our study is the first which evaluates HSP70 concentrations in peritoneal lavage fluid of children undergoing cholecystectomy. On the contrary, Braun et al. [32] found that among others IL-6 and HSP70 are parameters of early mRNA expression during intestinal ischemia-reperfusion injury in pigs. Expression of IL-6 mRNA increased at 2 hours after reperfusion and HSP70 at 3 hours after reperfusion. These results in general are consistent with our study, as we showed that HSP70 growth occurred in the first hours in response to surgical injury.

One of the study limitations could be a small number of children undergoing open cholecystectomy. However, it was due to that laparoscopic approach optimizes outcome, even though it has higher risk of bile duct injury than the classical open technique. Thus, the small number of samples obtained during open cholecystectomy reflects the tendency among surgeons to choose laparoscopic approach over the classical surgical cholecystectomy. Another study limitation is that we collected and preserved only peritoneal lavage fluid to assess early local response in the peritoneum to the surgical injury during the first hours. We did not measure serum IL-6. However, the main aim of our study was concentrated on the intraperitoneal immune response.

To conclude, our results demonstrated significant peritoneal lavage fluid IL-6 concentration growth measured at the end of the cholecystectomy as compared to the beginning, regardless of the type of the procedure. IL-6 growth during open cholecystectomy was greater compared to laparoscopic cholecystectomy. IL-6 concentration did not correlate with HMGB1 and HSP70, which indicate that the significant growth of this cytokine was not related only to mechanical tissue damage due to surgical procedure.

A clinical significance of the study could be related to the fact that the evaluation of IL-6 concentration in peritoneal lavage fluid by IVD test may be useful to assess an early local inflammatory response and help indicate individuals, which gain benefits from targeted intraperitoneal anti-IL-6 monoclonal antibody therapy. However, this aspect remains for further studies.

## Figures and Tables

**Table 1:** Characteristics of the patients directed to cholecystectomy.

	Study group (*N* = 37)		*p* value^∗^
The type of surgical approach	Laparoscopic (*N* = 23) (62%)	Open (*N* = 14) (38%)	

Gender (F/M)	Male (*N* = 14) (38%) Female (*N* = 23) (62%)		*p* = 0.823
	Male (*N* = 8) (35%) Female (*N* = 15) (65%)	Male (*N* = 6) (43%) Female (*N* = 8) (57%)	

Age	7-17 y (median, 13)		*p* = 0.521
	8-17 y (median, 12)	7-17 y (median, 14)	

Operation time	0.5 h-3.5 h		*p* < 0.05
	Up to 1.0 h (*N* = 9)	Up to 2.0 h (*N* = 10)	
	More than 1.0 h (*N* = 14)	More than 2 h (*N* = 4)	
	Lasting about 2 h (*N* = 12)	Lasting about 2 h (*N* = 10)	

Type of anesthesia	General anesthesia (*N* = 37) (100%)		

Blood loss (mL)	Nonsignificant	Nonsignificant	

Intra-abdominal pressure	12 mmHg	N/A	

Insufflation gas	CO_2_^∗∗^	N/A	

Intraoperative complications	None	None	

Type and dosage of medication	All patients after surgical approach received standard surgical care and postoperative treatment according to the standard treatment protocols of our clinic and after surgeries were given intravenous paracetamol^∗∗∗^		

Length of hospitalization after surgery	2-10 days (*N* = 37)		*p* < 0.05
	2-5 days (*N* = 23) (100%)	More than 5 days (*N* = 14) (100%)	

^∗^A *p* value < 0.05 is considered to show a significant difference between groups. ^∗∗^Warmed, humidified CO_2_ does not attenuate the early inflammatory cytokine response [39]. ^∗∗∗^Paracetamol has no influence on inflammatory response. N/A: not applicable.

**Table 2:** IL-6 concentration in peritoneal lavage fluid of children at the beginning and at the end of laparoscopic and open cholecystectomy.

IL-6 concentration (pg/mL)	Laparoscopic surgery		Open surgery	
	At the beginning	At the end	At the beginning	At the end
Median	**3.31**	**10.57**	**5.90**	**368.80**
Percentiles (25%-75%)	2.02-4.56	3.06-37.98	4.07-19.11	52.39-1007.00
*p* value^∗^	*p* = 0.004		*p* = 0.001	

^∗^A *p* value < 0.05 is considered to show a significant difference between groups (according to Wilcoxon matched pairs test).

**Table 3:** HMGB1 and HSP70 concentration in peritoneal lavage fluid of children at the beginning and at the end of laparoscopic and open cholecystectomy.

	Laparoscopic surgery		Open surgery	
	At the beginning	At the end	At the beginning	At the end
HMGB1 concentration (ng/mL)				
Median	**0.00** ^#^	**1.22**	**1.20**	**1.21**
Percentiles (25%-75%)	0.00-1.14	0.00-1.24	1.15-1.21	1.16-1.23
*p* value^∗^	*p* = 0.005		*p* = 0.563	
HSP70 concentration (pg/mL)				
Median	**0.00** ^#^	**10294.00**	**5806.00**	**15420.43**
Percentiles (25%-75%)	0.00-968.20	5192.00-11714.00	0.00-10729.00	11269.00-16141.00
*p* value	*p* = 0.000		*p* = 0.001	

^∗^A *p* value < 0.05 is considered to show a significant difference between groups (according to Wilcoxon matched pairs test). ^**#**^All values below the sensitivity of the ELISA test were considered as 0.00 ng/mL for HMGB1 and 0.00 pg/mL for HSP70.

**Table 4:** The statistical parameters of serum C-reactive protein (CRP) and white blood cell count (WBC) values measured 2 hours before and 8-10 hours after laparoscopic and open cholecystectomy.

	Laparoscopic surgery		Open surgery	
	2 hours before	8-10 hours after	2 hours before	8-10 hours after
CRP concentration (mg/L)				
Median	**1.0**	**7.1**	**0.6**	**67.4**
Percentiles (25%-75%)	0.5-2.6	5.6-9.1	0.5-1.9	53.4-76.3
*p* value^∗^	*p* = 0.000		*p* = 0.000	
WBC concentration (×10^3^/*μ*l)				
Median	**6.4**	**9.7**	**7.4**	**12.9**
Percentiles (25%-75%)	5.4-9.1	7.6-13.5	6.5-9.8	11.5-23.8
*p*-value∗	*p* = 0.038		*p* = 0.000	

^∗^A *p* value < 0.05 is considered to show a significant difference between groups (according to Wilcoxon matched pairs test).

## Data Availability

The datasets generated and analyzed during the current study are not publicly available but all are kept at the Medical University of Bialystok and available from the corresponding author on reasonable request.
